# Piezoelectric Energy Harvesting Controlled with an IGBT H-Bridge and Bidirectional Buck–Boost for Low-Cost 4G Devices

**DOI:** 10.3390/s20247039

**Published:** 2020-12-09

**Authors:** Daniel Teso-Fz-Betoño, Iñigo Aramendia, Jon Martinez-Rico, Unai Fernandez-Gamiz, Ekaitz Zulueta

**Affiliations:** 1 System Engineering and Automation Control Department, University of the Basque Country (UPV/EHU), Nieves Cano 12, 01006 Vitoria-Gasteiz, Spain; ekaitz.zulueta@ehu.eus; 2 Nuclear Engineering and Fluid Mechanics Department, University of the Basque Country (UPV/EHU), Nieves Cano 12, 01006 Vitoria-Gasteiz, Spain; inigo.aramendia@ehu.eus (I.A.); jon.martinez@tekniker.es (J.M.-R.); unai.fernandez@ehu.eus (U.F.-G.); 3 Automation and Control Unit, Fundación Tekniker, Basque Research and Technology Alliance (BRTA), 20600 Eibar, Spain

**Keywords:** piezoelectric, harvester, IGBT H-bridge, bidirectional buck–boost, low-cost 4G shield, supercapacitor

## Abstract

In this work, a semi-submersible piezoelectric energy harvester was used to provide power to a low-cost 4G Arduino shield. Initially, unsteady Reynolds averaged Navier–Stokes (URANS)-based simulations were conducted to investigate the dynamic forces under different conditions. An adaptive differential evolution (JADE) multivariable optimization algorithm was used for the power calculations. After JADE optimization, a communication cycle was designed. The shield works in two modes: communication and power saving. The power-saving mode is active for 285 s and the communication mode for 15 s. This cycle consumes a determinate amount of power, which requires a specific piezoelectric material and, in some situations, an extra power device, such as a battery or supercapacitor. The piezoelectric device is able to work at the maximum power point using a specific Insulated Gate Bipolar Transistor (IGBT) H-bridge controlled with a relay action. For the extra power supply, a bidirectional buck–boost converter was implemented to flow the energy in both directions. This electronic circuit was simulated to compare the extra power supply and the piezoelectric energy harvester behavior. Promising results were obtained in terms of power production and energy storage. We used 0.59, 0.67 and 1.69 W piezoelectric devices to provide the energy for the 4G shield and extra power supply device.

## 1. Introduction

Industry 4.0 and the Internet of Things (IoT), with data upload to the cloud, are common methods that aim to enhance systems by analyzing the measured data [1]. However, they oppose the worldwide need to reduce energy consumption to minimize the effects of climate change. As such, the use of energy harvesting systems appears to be one of the key solutions for reaching these objectives [2,3]. Energy harvesting refers to the use of ambient energy to power small electronic devices, such as sensors, microcontrollers, and health monitoring systems. The ambient energy is obtained using energy transducers, which apply different energy sources to produce enough energy to supply an electrical load. Since energy is produced by the energy transducer, the main advantage of energy harvesting is that no external power supply is needed, avoiding the need for a grid connection. This enables the use of sensors in remote or inaccessible areas in which no power supply is available [2,4].

Numerous ambient energy sources exist that can be used for energy harvesting, many of which are related to industrial applications such as vibrations, thermal gradient, noise, and air and water flow in pipes, among others [2,4]. Focusing on vibration-based energy harvesting (VeH), this energy source can be extracted using different techniques such as electromagnetic [5,6], electrostatic/triboelectric [7,8], or piezoelectric beams [9]. Among them, piezoelectric transducers provide several advantages, such as a high output voltage, high energy density and little mechanical damping [9].

Piezoelectricity is the property of some crystal, ceramic, and polymer materials to produce electricity when a mechanical strain is applied to them (direct piezoelectric effect) [10]. When an electrical field is applied to a piezoelectric material, it deforms; this is known as the converse piezoelectric effect. Taking advantage of the direct piezoelectric effect, a piezoelectric transducer can be connected to a vibrating element and produce electricity to feed a small load [4].

Several studies have proposed piezoelectricity-based solutions for energy harvesting purposes. Regarding solutions based on mechanical element vibrations, Wang et al. [11] proposed a piezoelectric energy harvesting generator for suspension structures, using a leaf spring with the piezoelectric layers connected to the workload. Wang et al. [12] developed another design for suspension structures using a piezoelectric stack and a force amplifier, obtaining a maximum peak-to-peak output power of 109 mW under a 0.3 g excitation at resonant frequency. He at al. [13] proposed a dual piezoelectric energy harvester driven by an inertial wheel. One of the piezoelectric transducers produced electricity by the deformation caused by the contact with a cover coupled to an inertial wheel. Another used the forces created by a magnetic system fixed to another piezoelectric beam. An energy conversion efficiency of 18.27% was obtained.

For flow-induced vibration-based solutions, both air [14,15,16] and water flows [17,18,19,20,21,22,23,24,25,26] can be exploited using piezoelectric transducers to produce electricity. Regarding water flow solutions, Allen et al. [17] and Taylor et al. [18] produced an energy harvesting eel, which consisted of a piezoelectric membrane placed in the wake of a bluff body to benefit from the generated von Kármán streets to produce electricity. Based on that idea, researchers recently tried to apply the same concept for water pipe systems, in which data measurements via energy harvesting are of interest. Yao et al. [19] performed some experimental simulations with an underwater flow-induced piezoelectric vibration energy harvester. The harvester consisted of a bluff body to which a piezoelectric vibrator was attached. Different bluff body shapes and diameters were tested in the study, the authors finding that circular geometry produced better results and that the output voltage was proportional to the diameter of the bluff body. Qureshi et al. [20] proposed a solution of various piezoelectric energy harvesters to monitor the critical parameters in the Turkey–Cyprus water pipeline. The solution was based in D-shaped bluff bodies, to which a flexible cantilever was connected in the wake of the D-shaped body. A piezoelectric film was located on the top of the cantilever, and, due to the von Kármán vortex streets, the cantilever was deflected and the piezoelectric device received mechanical stress, thus producing electricity. Zhou et al. [21] presented a tubular piezoelectric energy harvester and analyzed the influence of some parameters on the final performance: geometry, input mechanical load, and output electrical load. Following this work, Zhou et al. [22] performed a global stability analysis of the same harvester. They measured the efficiency of the system, and different simulations were conducted for providing guidelines for the practical design of an energy harvester. Cao et al. [23] designed a force-amplified stack energy harvester for hydraulic pipeline systems. The piezoelectric energy harvester took advantage of the pressure fluctuations in the pipeline caused by unsteady flows. A force amplifier connected to the pipeline by an interface film was used to amplify the external excitation force (caused by the pressure fluctuation) and deform the piezoelectric stack to produce electricity. Cottone et al. [24] developed and patented an energy harvesting system consisting of a piezoelectric beam connected to a cylinder-shaped oscillating body placed in a water pipeline. The piezoelectric transducer produced electricity due to the oscillations caused by the vortices generated in the wake of the cylinder. Aramendia et al. [25] presented a new control algorithm for the energy harvester proposed by Cottone et al. [24] using the cylinder lift coefficient, water velocity, and the piezoelectric voltage as control variables. Later, Aramendia et al. [26] presented a new design for the previous cylindrical-shaped body, proposing a U-shaped body, and proved that the novel geometry produces more power than the original design.

For the first time, we studied the design and application of an underwater energy harvester implemented in a water pipe to supply energy to a communication device that communicates data via 4G. This device uses piezoelectric material characteristics to provide the required energy. Therefore, an electronic circuit, which converts the piezoelectric Alternating Current (AC) power into Direct Current (DC), was designed. Not all the harvesters produce the necessary power to supply the low-cost 4G device during the communication. Another electronic circuit is needed to charge and discharge the supercapacitor or the battery. All the electronic calculations are based on 2D computational fluid dynamics (CFD) simulations of a semi-submergible piezoelectric energy harvester. The remainder of this paper is structured as follows: Section 2 describes the energy harvester system and the computational setup used for the numerical simulations. Section 3 outlines the development of the electronic circuit. An adaptive differential evolution JADE-based algorithm for energy harvester power optimization is presented in Section 4 and all the results are provided in Section 5. Finally, the main conclusions are summarized in Section 6.

## 2. Harvester Description and Computational Setup

**Figure 1:**
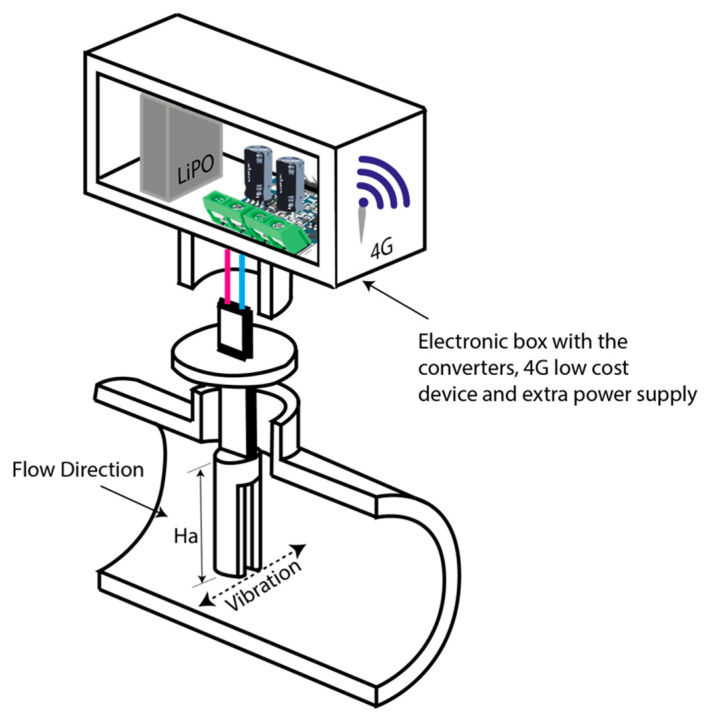
Water pipe with the U-shaped geometry assembled with the energy harvester as the oscillating body (not to scale). Ha—the length of the oscillating body.

The harvester studied in the present work is directly related to the system developed by Cottone et al. [24]. This mechanism contains a piezoelectric beam assembled to an oscillating body that is introduced within water pipelines 2–5 inches in diameter, as illustrated in Figure 1.

**Figure 2:**
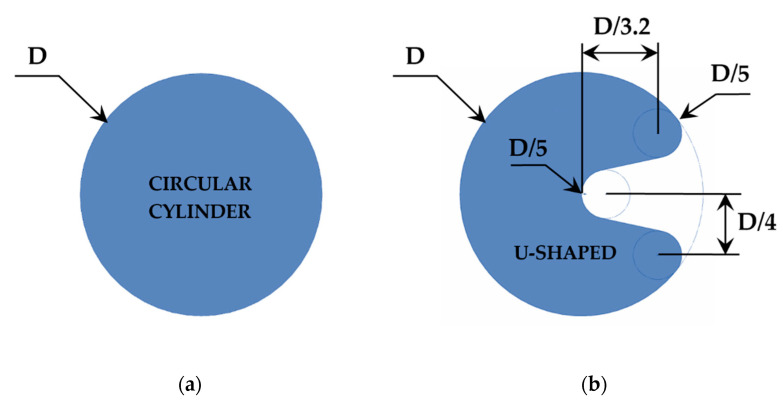
Sectional representation of the oscillating body: (**a**) circular cylinder and (**b**) U-shaped geometry.

Vortices in the area nearly behind the oscillating body are generated due to the vibrations produced by the impact of the water. Two different geometries were evaluated as the oscillating body, a circular cylinder geometry and an innovative U-shaped geometry, to optimize the extraction of kinetic energy from the water incoming through the water pipe. These proposed geometries are based on the CFD simulations performed previously by Aramendia et al. [25,26]. The shape and dimensions of each geometry are presented in Figure 2.

In the current work, the commercial code STAR CCM+ (v. 14.02.011, Siemens) [27] was used to develop and characterize the numerical model for the semi-submergible harvester with both oscillating bodies. Regarding the computational domain, a total length of 50 times the body diameter (D) was applied to accurately study the vortices formed behind the oscillating body. Diameters of 10, 20 and 30 mm were studied for both geometries. A velocity inlet was applied to achieve the different Reynolds numbers (Re) considered in this study (3000, 6000, 9000 and 12,000). Re is a dimensionless number based on the oscillating body diameter (D) and was obtained using

(1) Re = Vwater × D × ρwaterµ

where $\rho_{\mathrm{water}}\;$ and µ correspond to the density and dynamic viscosity of water at 15 °C, respectively.

A pressure outlet condition was applied to the boundary located downstream of the oscillating body and a slip condition for both top and bottom wall boundaries. The mesh generated consisted of 2D polyhedral cells, mostly located in the region behind the oscillating body with the generation of fully anisotropic wake refinement. A volumetric control was defined around the body to refine the mesh near to the wall and to maintain the dimensionless wall y^+^ < 1. This mesh was chosen after performing the mesh dependency study used by Aramendia et al. [25] to verify that the solution obtained was independent of the mesh resolution.

In the present study, the numerical solution of the unsteady flow was obtained applying the Reynolds averaged Navier–Stokes (RANS) equations. An upwind scheme [28] was used to discretize the convective terms, ensuring the robustness of the solution, and the turbulence was modelled with the k–ω shear stress transport (SST) turbulence model developed by Menter [29]. A time-step (Δt) of 0.002 s was defined in all the numerical simulations presented in this work with 15 inner iterations to accurately capture the vortex shedding formed closely behind the oscillating body. The temporal discretization order can also affect the accuracy of the solution. In this work, where aerodynamic unsteady shedding simulations were studied, a second-order time discretization was applied. A solution time of 20 s was simulated, and simulations were considered converged when satisfactory residuals were achieved in terms of pressure, turbulence, and velocity quantities.

## 3. Electronic Circuit Development

For the simulation, the AC-/C piezoelectric energy harvesting circuit of Covaci et al. [9] was implemented. The electronic consumption was estimated to determine that the harvester was able to provide the necessary power. We used low-cost devices to estimate that power. From the analysis of all piezoelectric devices and due to the electronic power consumption, three piezoelectric devices were implemented into the circuit.

The low-cost 4G device uses about 400 mA when communicating and 100 mA in power-saving mode. Therefore, the power consumption is 1.32 W in communication mode and 0.33 W in power-saving mode. We considered that the control electronics use about 0.075 W. An additional 20% margin was considered for power loses. The communication uses 1.7 W to communicate the data and 0.5 W when in power-saving mode:

(2) $$P_{\mathrm{comunication}}\;=\left(1.32\;W+0.075\;W\right)\cdot \frac{120\;\%}{100\;\%}=1.7\;W$$

(3) $$P_{\mathrm{Saving}}\;=\left(0.33\;W+0.075\;W\right)\cdot \frac{120\;\%}{100\;\%}=0.5\;W$$

**Figure 3:**
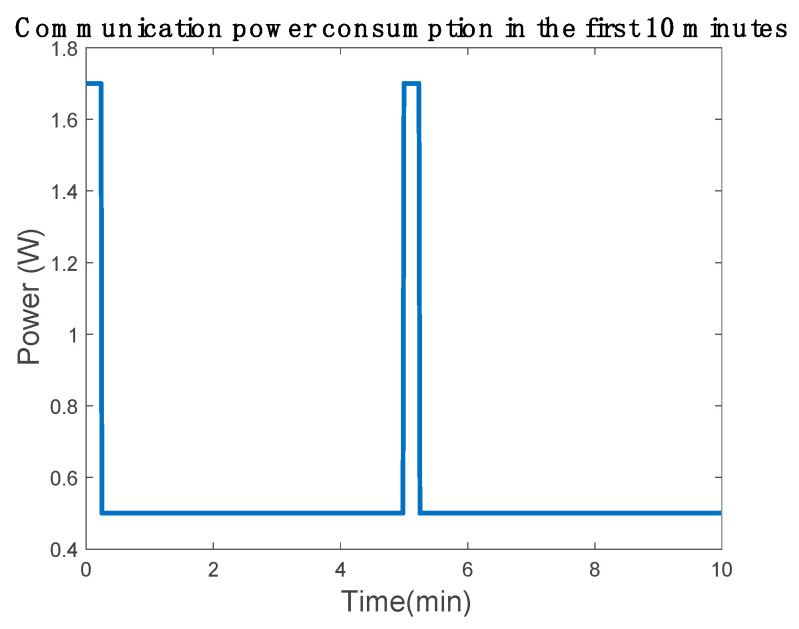
Communication device power consumption representation.

To calculate the battery capacity, it was necessary to set the duration for which the device sends and receives data. The data bytes were sent every 5 min, in which the device remained in power-saving mode more than 4 min and 15 s in data sending mode, as shown in Figure 3. Consequently, the power consumption per hour was 0.56 Wh. This value helped to determinate the piezoelectric device characteristics by discarding the configurations that do not generate enough power to supply the system.

Along with the power consumption, the determination of energy storage to power the Arduino in communication mode is a key issue. There are two conventional alternatives: LiPO batteries and a capacitor. The nominal voltage of the LiPO battery is 3.7 V; to determinate the battery capacity, we used one-cycle power consumption. A battery has to supply the necessary power to supplement the power from the energy harvester. In the present work, the piezoelectric power during the communication was set to 0 to oversize the battery in order to determine the capacity. The power consumption (E) was estimated during one cycle of the communication mode in one hour using Equation (4), and the battery capacity (C) was calculated using Equation (5) considering a depth of discharge (DoD) of 60%. The maximum battery capacity must be 3.1 mAh and, depending on the device power capacity, the battery size can be less than the estimated value. However, this capacity is unusual in the market so we used a 15 mAh battery for the simulation, which is a commercial capacity.

(4) $$E_{\mathrm{power}}\;=1.7\;W\cdot \frac{15\;s}{3600\;s}=0.007\;\mathrm{Wh}$$

(5) $$C_{\mathrm{Battery}}\;=\frac{\frac{0.007\;\mathrm{Wh}}{3.7\;V}}{60\;\%}=0.0031\;\mathrm{Ah}$$

Equation (6) was used to calculate the capacity of the supercapacitor, achieving a result of C = 1.68 F, corresponding to a 5.5 V supercapacitor, as shown in Equation (7). Considering the commercial components used, the capacity was determined as 2.5 F.

(6) $$E_{C}=\frac{1}{2}\cdot C\cdot V^{2}$$

(7) $$C=\frac{1.7\;W\cdot 15s\cdot 2}{{\left(5.5\;V\right)}^{2}}=1.68\;F\;$$

**Figure 4:**
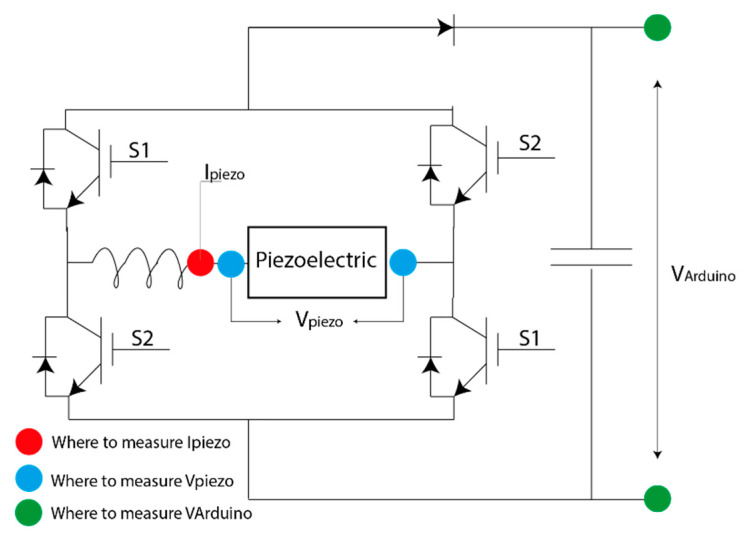
Electronic circuit that works at the maximum power point.

An electronic circuit was implemented to work at the maximum power point of the energy harvester. In the current work, an IBGT with diode H-bridge was selected as an AC/DC converter device. An inductor was set in the H-bridge entry and a diode in the output to work as a boost (Figure 4). Thus, the selected electronic circuit is an AC/DC boost.

**Figure 5:**
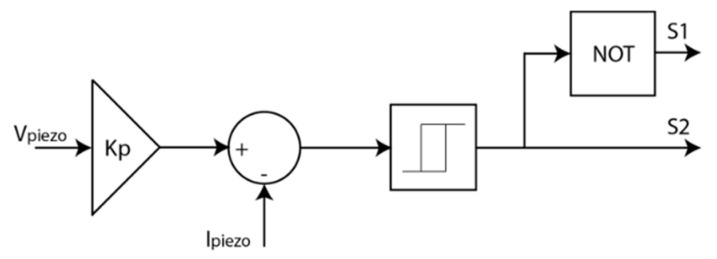
Control design for the piezoelectric system.

S1 and S2 are opposite each other and the switching is controlled via a relay; when the error is negative, the relay output is 1 and 0 when the error is positive. The control is defined by the current with the previously calculated Kp to convert the piezoelectric voltage into current (Figure 5).

**Figure 6:**
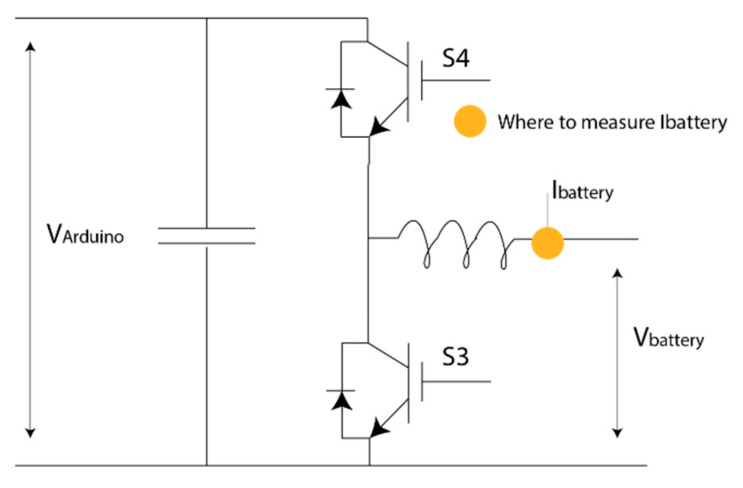
Electric circuit for the battery to provide the power to the Arduino bus.

To maintain the V_arduino_ at 5 V, an additional electronic circuit was necessary. A bidirectional buck–boost converter was chosen, as shown in Figure 6. Therefore, the battery was able to provide power to the Arduino bus and can be charged when the Arduino is in power-saving mode.

**Figure 7:**
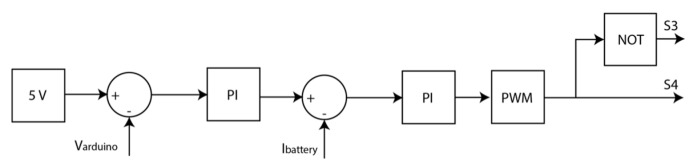
Control design for the piezoelectric system.

S3 and the S4 are opposite each other and the switching is controlled via two Proportional Integrator (PI) in cascade (Figure 7). The first PI tries to control the low-cost bus voltage (V_Arduino_) and the output of the controller is limited to ±0.2 A. This limitation controls the battery discharge capacity. In the second PI controller, the output of the controller was set to 0 to 0.95, so this output was converted to a 5 KHz Pulse With Modulate (PWM) signal.

The values of the different electronic passive components and PI controller values are summarized in Table 1.

To conduct the simulation, the low-cost power consumption was modeled. The low-cost device in power mode without electrical losses consumed about 1.4 W and in power-saving mode 0.4 W. The energy consumption was modeled by implementing a variable resistor connected in parallel in the low-cost bus. Therefore, if the low-cost device needs 5 V to power up, these values are 3.57 Ω for communication and 12.5 Ω for power saving, which will change with the communication cycle, as shown in Figure 3.

## 4. JADE-Based Algorithm for Power Optimization

The JADE algorithm, first introduced by Zhang et al. [30] was used as the optimization algorithm to maximize the power extracted from the harvester. It consists of a variation of the differential evolution (DE) algorithm, which provides improvements in the convergence and diversification of the population through its execution. Aramendia et al. [25] described the model of the dynamics of a semi-submergible piezoelectric energy harvester system. The selection of the optimal parameters during the harvester design process enables maximizing the power generated by the harvesting system. Equation (8) was used to calculate the extracted power:

(8) $$p\;=\;{\left(\frac{\alpha .a}{K_{\mathrm{trans}}}\right)}^{2}\frac{{\dot{\theta }}^{2}}{K_{p}}={\left(\frac{\alpha .a}{K_{\mathrm{trans}}}\right)}^{2}\frac{1}{K_{p}}{\left(\frac{a_{1}w_{0}{.C}_{L.\max}}{\sqrt{{\left(a_{4}{\;-\;a}_{2}w_{0}{}^{2}\right)}^{2}\;+\;{\left(a_{3}w_{0}\right)}^{2}}}\sin\left(w_{0}t\right)\right)}^{2}$$

Then, the mean value of the instantaneous power was obtained over the period given by an angular pulsation of the lift coefficient$\;w_{0}$:

(9) $$p_{\mathrm{mean}}={\left(\frac{\alpha .a}{K_{\mathrm{trans}}}\right)}^{2}\frac{1}{2{.K}_{p}}{\left(\frac{a_{1}w_{0}{.C}_{L.\max}}{\sqrt{{\left(a_{4}{\;-\;a}_{2}w_{0}{}^{2}\right)}^{2}\;+\;{\left(a_{3}w_{0}\right)}^{2}}}\right)}^{2}$$

The model parameters and variables used in the control of the energy harvester are described in Table 2 and Table 3, respectively.

## 5. Results

### 5.1. Computational Results

**Figure 8:**
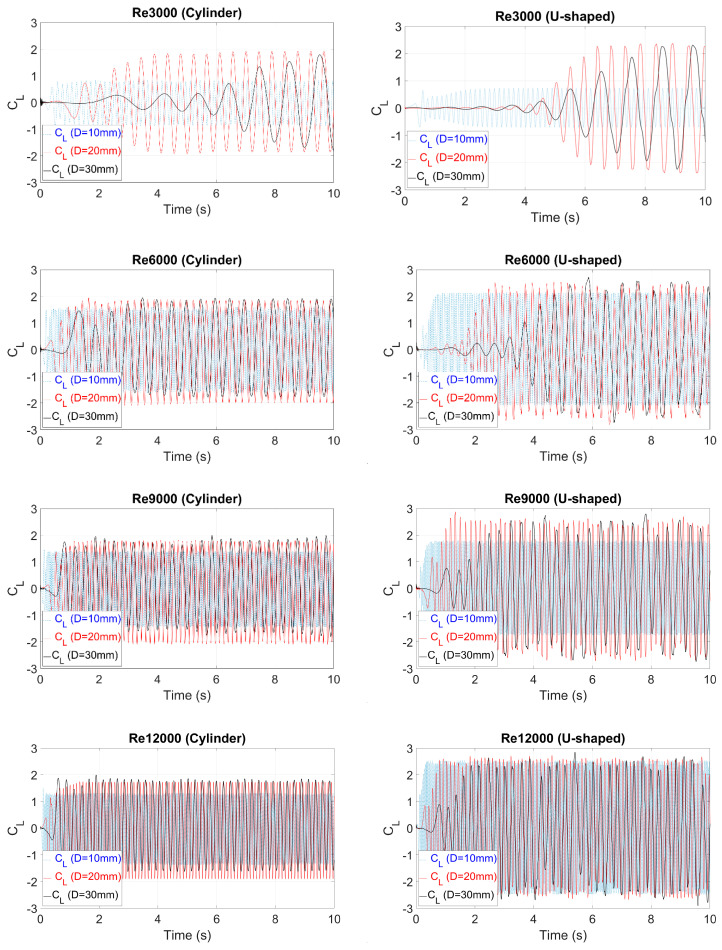
Evolution of the lift coefficient (C_L_) for the circular cylinder and U-shaped geometries at each Reynolds number.

The evolution of the lift coefficient (CL) at each Re and oscillating body diameter investigated is shown in Figure 8 for the circular cylinder and U-shaped geometries. This dimensionless coefficient was obtained using Equation (10), where F_L_ represents the force perpendicular to the flow direction caused by the water in the oscillating body.

(10) $$C_{L}\;=\;\frac{F_{L}}{0.5{\;\times \;\rho }_{\mathrm{water}}\;{\;\times \;U}_{\infty }^{2}\;\times \;D}$$

### 5.2. Electronic Circuit Results

Having defined both the electronics and the extra power supply components, the results of the electronic simulations are presented in this section. The JADE optimization provided different power results, but only those harvesters that provided the necessary energy to recharge the extra power supply and to feed the low-cost communication device were studied. The minimum energy value to be provided by the energy harvester was 0.56 W; therefore, we used only three cases to test the electronics. The first case provided around 0.5908 W, the second one 0.6799 W, and the last one 1.6998 W (Table 4). However, the 1.6998 W piezoelectric device had enough power to provide the necessary energy to the Arduino bus. The parameter Ha presented in Table 4 corresponds to the length of the oscillating body.

The harvester that provided 0.5908 W was considered the lowest power device. This device produced 0.03 W more than the calculated limitation. During communication mode, the piezoelectric harvester and the battery or supercapacitor provide the necessary power to maintain the Arduino bus voltage. However, in power-saving mode or flying mode, the piezoelectric device provides the necessary power to maintain the Arduino bus voltage and to recharge the battery or supercapacitor.

**Figure 9:**
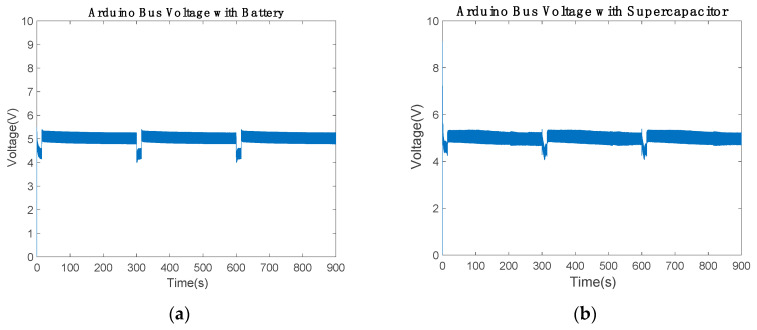
Low-cost bus voltage over 900 s using the (**a**) battery and (**b**) 2.5 F supercapacitor.

Figure 9 shows how the battery and the supercapacitor have enough energy to maintain the Arduino bus around 5.5 and 4.3 V to maintain the acceptable performance of the low-cost communication device. The supercapacitor performed slightly better than the battery since the Arduino bus maintained higher voltage in communication mode (Table 5).

**Figure 10:**
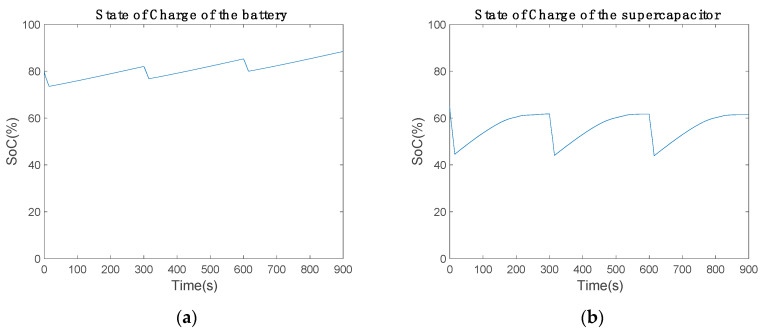
State of charge of the power supply over 900 s using a (**a**) battery and (**b**) 2.5 F supercapacitor.

Figure 10 shows the state of charge (SoC) of the battery and supercapacitor. The battery started with 80% and discharged about 5% in communication mode. In power-saving mode, the battery recharged 8%. The extra power device gains 3% of the SoC per cycle (Figure 10a). Thus, the battery will be fully charged and the extra 3% has to be used. However, the supercapacitor behaves differently in charge and discharge cycle. The maximum SoC is 61.79% and the minimum is 44.07%, which are constant for each communication cycle. Therefore, the performance of the supercapacitor is suitable for the current electronic circuit.

**Figure 11:**
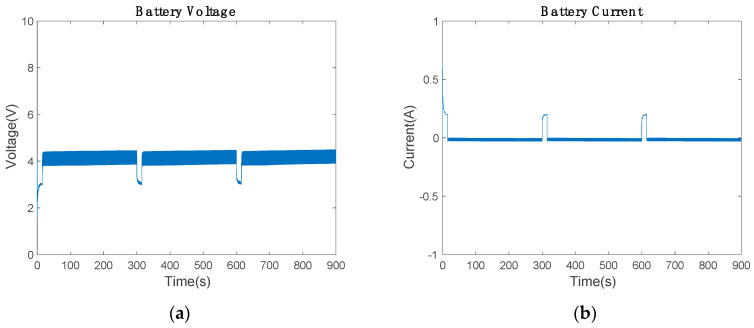
Battery state during the communication: (**a**) voltage and (**b**) current of the battery.

To evaluate the battery performance, the voltage was investigated, as shown in Figure 11a. When the Arduino starts the communication, the battery voltage drops to provide the necessary energy in the bus. This voltage cannot be lower than 2.75 V, since the battery cuts off the power to prevent cell damage. However, the results showed that the battery stays in the range of 3 to 4.4 V. The battery in communication mode discharges 0.2 A and charges with 0.02 A (Figure 11b). The discharge current adopts that value due to the current PI. Therefore, the current controller prevents battery energy cut-off and controls the maximum current discharge and charge values.

**Figure 12:**
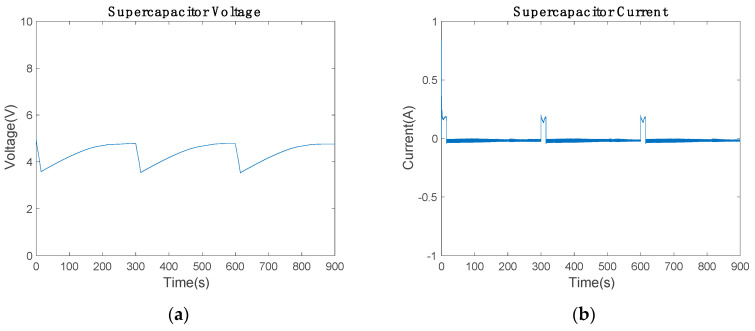
Supercapacitor’s state during the communication: (**a**) voltage and (**b**) current of the supercapacitor.

Figure 12 shows the supercapacitor voltage and current, which are similar to those of the battery. The current controller PI discharges the supercapacitor controlling the current between ±0.2 A. The maximum discharge current is 0.2 A and the supercapacitor voltage varies from 3.566 to 4.771 V.

Figure 11a and Figure 12a show the initial conditions for the electronic simulation. The battery starts with 80% of SoC and the supercapacitor with 5 V. During the whole electronic circuit simulation, the average power of the piezoelectric device remains constant. Hence, the proposed Insulated Gate Bipolar Transistor (IGBT) H-Bridge works satisfactory as AC/DC booster, which separates the input from the output. Consequently, the piezoelectric device works in the maximum power point in all situations, although the low-cost communication device alters the Arduino bus resistance depending on which mode (communicating mode or power-saving mode) is used.

The medium-power energy harvester generated 0.6799 W and 0.6153 V. The resultant power was higher than that obtained with the minimum-power device. This means that the battery charged faster and needed variable resistance to reduce the extra power of the bus. For the current harvester, only the supercapacitor was used to display the results.

**Figure 13:**
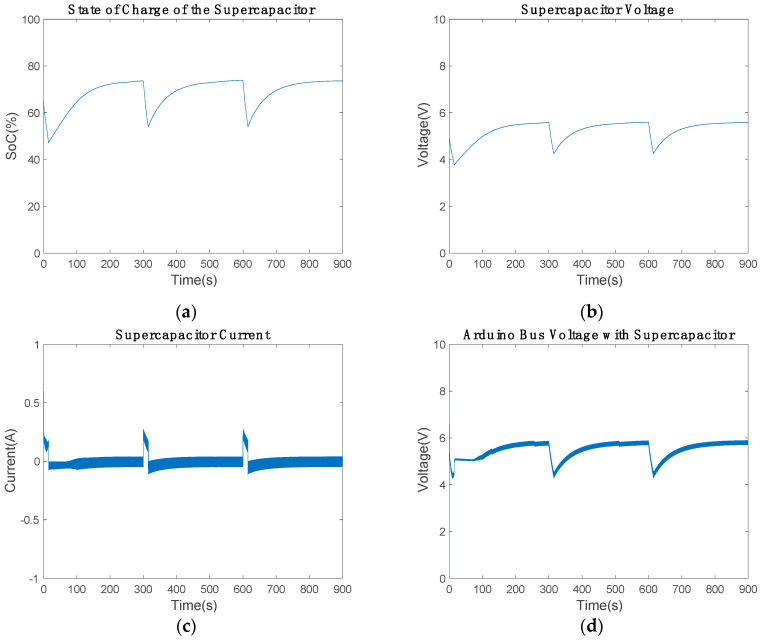
Medium-power harvester results: (**a**) state of charge during 900 s, (**b**) voltage of the supercapacitor, (**c**) current of the supercapacitor, and (**d**) the low-cost bus voltage.

The medium-power piezoelectric energy harvester behaved similarly to the minimum-power device. However, the SoC revealed that the supercapacitor stored more energy (Figure 13). In this second simulation, the SoC stayed between 54.58% and 73.87%, which is about 12% extra energy in comparison with the minimum-power device. This extra power increased the low-cost bus voltage to 5.54 V in communication mode, which was also visible in power-saving mode, as shown in Table 6. However, these voltage values are acceptable for the Arduino, and the modification of any electronic component was not required.

The maximum-power piezoelectric device provided by the energy harvester was 1.6998 W, corresponding to a voltage of 1.1859 V. This device has enough power to supply the low-cost communication device in communication mode. Hence, the energy of the battery or supercapacitor was not implemented in the electronic circuit.

**Figure 14:**
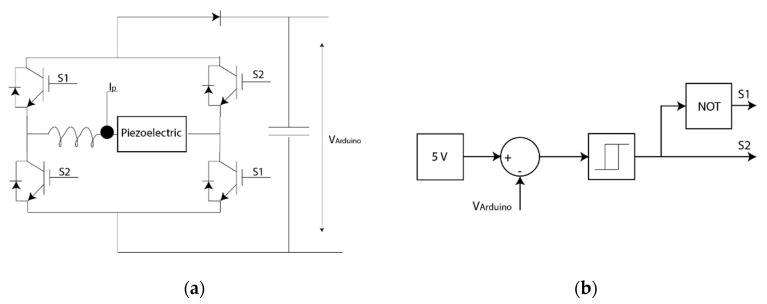
Electronic circuit for the maximum power: (**a**) electronic circuit and (**b**) relay control of the voltage of the low-cost bus.

In power-saving mode, the extra power devices charge until their maximum SoC. This harvester needed an additional electronic circuit to control the energy harvester maximum power point (Figure 14), where the IGBT H-bridge controlled the bus voltage. The relay controlled the IGBT switching by considering the error of V_arduino_ and 5 V. If V_arduino_ is higher than the reference, the S2 value is zero.

**Figure 15:**
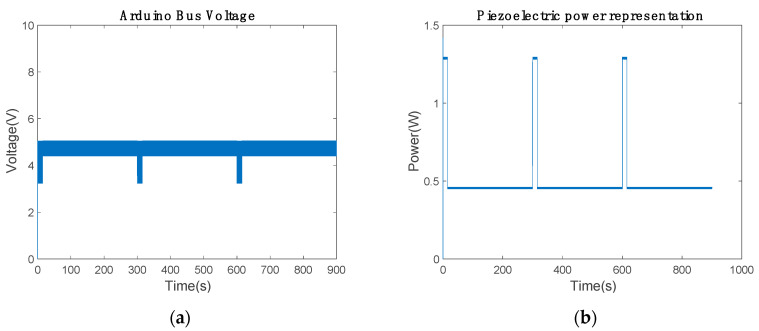
Maximum power of the energy harvester: (**a**) low-cost 4G shield bus voltage and (**b**) piezoelectric mean power.

The low-cost 4G bus voltage is shown in Figure 15a. The mean voltage was 4.4175 V in communication mode and 4.833 V in power-saving mode (Table 7). Thus, it had enough voltage to power up and communicate the values. The proposed controller presented in Figure 14b needed an integrator to maintain the bus voltage near to the reference. Additionally, the piezoelectric power had to be lower than 1.6998 W; in the results, the power remained below 1.3 W, as illustrated in Figure 14b.

The proposed IGBT H-bridge also worked to control the low-cost device when the device has the necessary energy to supply the electronic components in communication mode. To stop the electronic simulation, the inductances were adjusted to optimize the electronic components. The inductance in Figure 4 was reduced to 470 μF and that in Figure 6 to 800 μF. These values guaranteed the performance of the electronic circuit.

**Figure 16:**
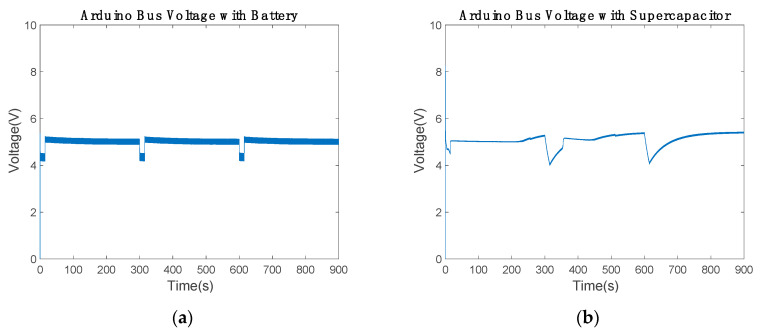
Arduino bus voltage with inductance minimization: (**a**) using the battery and (**b**) using the 2.5 F supercapacitor.

The Arduino bus voltage with battery was maintained in the range between 4.3 and 5.03 V (Figure 16a and Table 8). Therefore, the results were not modified by the variation in the inductance. According to Figure 9a, the voltage of the Arduino bus was modified. In communication mode, both results were the same. However, the bus voltage decreased from 5.5 to 5.03 V in power-saving mode with inductance variations. The Arduino bus voltage with the supercapacitor ranged from 4.2 to 5.1 V. When the supercapacitor was about 65% of capacity, the voltage was close to 5 V. Therefore, in the time intervals of 250 to 300, 450 to 600 and 700 to 900 s in Figure 16b, the Arduino bus voltage increased. Note that the 800 μF inductance cannot be reduced; hence, the supercapacitor was unable to provide the necessary power to maintain the Arduino bus voltage in the reference.

**Figure 17:**
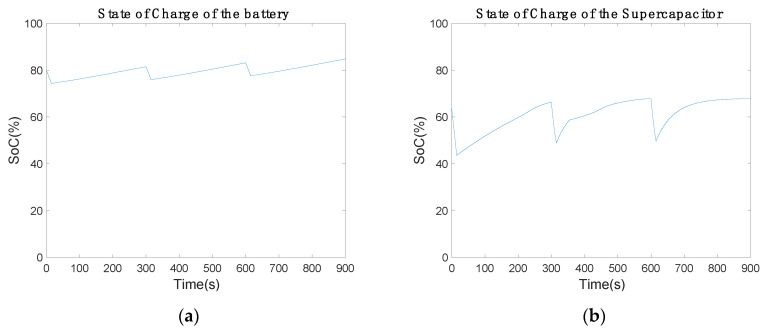
State of charge of the power supply with inductance minimization: (**a**) using the battery and (**b**) using the 2.5 F supercapacitor.

Figure 17a represents the battery SoC with new inductance values. The battery losses were about 5% of each capacity in communication mode, since the battery provided the necessary power for the low-cost communication device. In power-saving mode, the battery recharged about 7.07% of each SoC. In comparison with the previous results, the recharge capacity was reduced by about 1% (Figure 10a). Thus, a slight modification in the electronic circuit behavior was introduced by the inductance minimization. Figure 17b shows the supercapacitor SoC. The supercapacitor discharged about 17.45% of each capacity in communication mode to provide enough power to the Arduino bus. In power-saving mode, the supercapacitor gained 19% of each capacity. By comparing Figure 10b with Figure 17b, the inductance minimization behavior can be observed. In Figure 10b, the supercapacitor achieves a capacity of 61.7% and stops charging. Similarly, Figure 17b shows how it achieves a capacity of 67.84%. Therefore, the supercapacitor charge was improved, charging the supercapacitor more than 61.7%.

Finally, this minimization did not significantly alter the circuit behavior. It has the necessary power to communicate and to recharge the extra power device. The Arduino bus maintained the voltage between 5.5 and 4 V, which is necessary for the microcontroller.

## 6. Conclusions

The proposed electronic circuit works well with these piezoelectric energy harvesters, which provide the minimum power for the electronics. In the present work, the power was set to 0.56 W due to the electronic device and the communication cycle that was selected. Normally, this application requires devices with low energy consumption to use other piezoelectric devices and different pipeline diameters. In the present work, more than 10 inches of pipeline was used.

As per the results, the harvester provided more energy than estimated, needing extra resistance to lose energy when the battery or supercapacitor was fully charged. To not lose that energy via heat, it is better to use a supercapacitor rather than a battery. If the piezoelectric power is the same or higher than the estimated maximum power consumption, the device only needs the IGBT H-bridge by controlling the low-cost 4G bus voltage. In this situation, it is better to use the PI controller to minimize the bus voltage error.

Notably, the H-bridge works as an AC/DC boost, which separates the input from the output. Thus, it can connect any resistance at the output without altering the input. The bidirectional buck–boost also provides the necessary power for the low-cost communication device in both modes. When the communication mode is activated, it helps to maintain the voltage; when in power-saving mode, the extra power device will charge.

In future work, these electronics will be tested in a real situation, in which the piezoelectric energy harvester will provide the necessary power to communicate data.

## Figures and Tables

**Table 1:** Electronic passive components and Proportional Integrator (PI) adjustment.

Component	Value
Figure 4 Inductance	1 mH
Figure 4 Capacitor	1000 μF
Figure 6 Inductance	10 mH
Figure 6 Capacitor	100 μF
Figure 7 PI voltage controller proportional value	1
Figure 7 PI voltage controller integral value	0.01
Figure 7 PI current controller proportional value	1
Figure 7 PI current controller integral value	1

**Table 2:** Model parameters.

Variable	Definition	Value	Unit
a	Force application distance point	0.01	m
C	Piezoelectric capacitance	1	nF
f	Frictional coefficient	0.01	(N·m·s)/rad
Ktrans	Transduction gain	2	-
α	Voltage induced bending factor	100	A s/m
$\rho_{\mathrm{water}}\;$	Fluid density	997.5	kg/m^3^

**Table 3:** Model variables.

Variable	Definition	Unit
C_L,max_	Maximum lift coefficient	-
J_wt_	Oscillating body inertia moment	kg·m^2^
Kp	Proportional gain	A/V
Kspring	Spring constant	N/m
t	Time	s
T_m_	Moment generated by the piezoelectric beam	N·m
T_Hydro_	Hydro-mechanical torque	N·m
u_1_	Reference of the piezoelectric deflection	m
V_1_	Piezoelectric voltage	V
$\omega_{0}$	Angular pulsation of the lift coefficient	rad/s
θ	Beam angle	rad

**Table 4:** Summary of the energy harvester characteristics.

Name	Cylinder or U-Shaped D (mm)	Ha (mm)	Power (W)	Voltage (V)	Kp (A/V)
Minimum power	10	254	0.5908	0.6969	44,686
Medium power	20	254	0.6799	0.6153	6374
Maximum power	10	254	1.6998	1.1859	44,641

**Table 5:** Summary of the minimum-power piezoelectric harvester results.

Component	Value
Mean voltage in communication mode with battery	4.35 V
Mean voltage in power-saving mode with battery	5.03 V
Mean piezoelectric power with battery	0.5962 W
Mean voltage in communication mode with supercapacitor	4.54 V
Mean voltage in power-saving mode with supercapacitor	5.02 V
Mean piezoelectric power with supercapacitor	0.5961 W

**Table 6:** Summary of the medium-power piezoelectric harvester results.

Component	Value
Mean voltage in communication mode with supercapacitor	5.01 V
Mean voltage in power-saving mode with supercapacitor	5.54 V
Mean piezoelectric power with supercapacitor	0.6752 W

**Table 7:** Summary of the maximum power piezoelectric harvester results.

Component	Value
Mean voltage in communication mode	4.42 V
Mean voltage in power-saving mode	4.83 V
Mean piezoelectric power in communication mode	1.29 W
Mean piezoelectric power in power-saving mode	0.45 W

**Table 8:** Summary of the minimum-power piezoelectric harvester results.

Component	Value
Mean voltage with battery in communication mode	4.36 V
Mean voltage with battery in power-saving mode	5.03 V
Mean piezoelectric power with battery	0.5936 W
Mean voltage with supercapacitor in communication mode	4.43 V
Mean voltage with supercapacitor in power-saving mode	5.04 V
Mean piezoelectric power with supercapacitor	0.5937 W
